# The Hospitals and the Public

**Published:** 1887-10-29

**Authors:** Baron Ferdinand de Rothschild

**Affiliations:** President and Founder of the Evelina Hospital for Sick Children


### The Hospitals and The Public.

By Baron Ferdinand de Rothschild, M.P.,
President and Founder of the Evelina Hospital for Sick Children.

At the outset let us consider the urgent need which exists for raising what at first sight appears to be a large, but what is in reality a small sum of money, £80,000, in order to cover the necessities of the hospitals of London. I am appalled that such strenuous efforts should be required, in this great city, to raise so paltry a sum, when I consider the wealth of this country and the munificence of the inhabitants. The sum is, and ought to be, considered a small sum for so great, so noble, so necessary an object. It has always been the pride of the British nation to rely upon themselves in cases of emergency, and not upon the State. In foreign countries hospitals—and the play-houses as well—are subventioned by the State. Here the hospitals are dependent upon the generosity of the public, and maintained by private and voluntary contributions. The fact stares us in the face that during the past few years the income of most of our hospitals has fallen off.

If we inquire into the causes of this falling off we may perhaps arrive at the remedies to apply in order to raise the sum necessary to ensure the future efficiency of our institutions. There are several causes. One reason which must necessarily affect the incomes of the hospitals is the existing agricultural depression in the country. This must do much to lessen the incomes of charitable institutions, for the simple reason that the incomes of the donors are lessened. With regard to industrial depression, this, I think, is somewhat exaggerated. However great it may be over the country, I deny that the industrial depression of London affects the incomes of the hospitals. When I see the magnificent shops at the West-end, and the people who frequent them, I say that the industrial classes of London are in a position to contribute, largely and handsomely, to the maintenance of our hospitals. But with agricultural depression the case is different. Take Guy's Hospital, for instance. Its chief income is derived from land. The proprietors of this land, who are the trustees of the hospital, have had to remit 40 per cent. to their tenants. The funds of the hospital must, of course, suffer in consequence. Great as is the philanthropic spirit, great and excellent as are the feelings of charity and benevolence throughout the country, the spirit of charity has found its way into somewhat different channels from those in which it used to flow. We have new movements, such for instance as the temperance movement. I am not for one moment speaking against this or any other movement, but what I say is, that they divert the flow of public charity from hospitals. Then there are free libraries; you cannot expect that, if a man chooses to establish a free library, he will give the same amount of money to hospitals in the future as he has done in the past. Then there has been a greater tendency of late to cultivate the mind, and to think more of our mental and moral improvement than of our physical ailments. We get a proof of this overculture in the army. Look at the examinations which now take place to qualify a man to become an officer. In former times the only qualifications were pluck and health. Now he has to solve difficult problems in mathematics, and if he does not he is plucked—a *pluck* of another kind. There is perhaps a good side to this, arising from the fact that the German students have frequently to wear spectacles. Their bad eyesight has been the means of producing good oculists in Germany—oculists who are the greatest in the world.

There are also other reasons which have tended to decrease the incomes of the hospitals. There has been a tendency of late to establish small special hospitals instead of supporting large general hospitals. I ask all present whether it does not stand to reason that a small hospital costs relatively more to keep than a large one. The waste must proportionately be considerably greater in a small than in a large hospital. I am fully aware that most members of the committee are unpaid, but you have the officers and other paid servants. Even from a medical point of view small special hospitals are a mistake. How, for example, can students rush from one end of London to the other? The same applies to physicians and surgeons. What I would urge is, give to special diseases and illnesses special wards in general hospitals. If anyone wishes to perpetuate his name, I would say to him, For God's sake do not erect a special hospital, but leave your money for the erection of a ward or the foundation of a cot in a general hospital.

There is one item connected with hospitals which ought to be considered, and it is this: there are in several hospitals of London very highly paid officials—the treasurers. I do not wish to allude to any one particular case, but there are some treasurers who receive enormous salaries. They do undoubtedly most excellent work, but this could just as efficiently be done by a lawyer, with a small salary. I must also allude to the expenditure entailed by the houses—in fact, in one case, the palace—which are afforded as the domain of those happy treasurers. What we want is not a great number of hospitals, but a concentration of means. During last year the voluntary hospitals of London relieved as many as 1,003,000 cases, at a total cost of £532,000, which exceeded the ordinary income of those hospitals by nearly £100,000. This number of one million individuals—for we need not trouble about the odd three thousand—represents a quarter of the population of London. At first sight it might appear awful to think that one million people, that is, 25 per cent., had to seek medical advice at the hospitals out of a population of four millions, and it might lead you to the belief that the sanitary arrangements of London were bad. But how many of you have not, during the past year, had a doctor's bill of your own to pay? The poor, the destitute, the needy, who live in hovels or wretched dwellings, think what it is to them to be allowed to enter one of those palaces on the south side of the Thames, and to there receive a warm bed, fuel, good food, and not only these physical advantages, but the kind, the genial words of the doctor, and the attentions of the skilled nurses. It is for these poor people that we appeal to you that they should not be refused admission to these palaces of mercy which our ancestors so munificently raised.

As I have said, it appears at first sight as if the condition of London were not in a satisfactory state, but just the contrary is the case. After comparing the mortality of London with other cities, not of England only, but of the Continent, I have no hesitation in saying that the condition of the metropolis is one of better sanitation than most towns throughout the world. This fact is itself largely due to the existence of our hospitals, where the science there developed has gone towards perfecting the condition of our city. Hospitals have from time immemorial existed, but formerly they were hot-beds of fever. Now they are just the reverse. They prevent the spread of those horrible epidemics which have decimated England in former times—such as the Great Plague in the time of Charles II—and other illnesses which still ravage the Continent. Take the cholera, for instance, which has advanced like a fiend to our doors, but which, during the past twenty years, has stopped short at the Channel, because it knew it would have no ground on which to sow its foul seeds. The popularity of hospitals among the poor has become great. Formerly they had a sort of dread—a dislike to going into hospitals. Now, owing to the excellent cures which have been made, and the principles which have been diffused, the hospitals are very popular.

I may say that I have seen with some regret that the press has not taken up the Hospital Sunday movement with more warmth and kindness. When I see that columns and columns are devoted to the political questions of the day, I regret that the press has not considered it worth its while to devote a few more words to this great hospital question. To-day I have carefully looked over five of the leading morning journals to see what account had been given in these papers of the great hospital meeting held last June under the presidency of the Archbishop of Canterbury at Lambeth Palace. In two it was entirely ignored, and in the other three there were only short accounts. If the editors of these papers had only put in a few telling sentences about these meetings, great good might have been done, and an abundance of support given to the hospitals. Personally I am ambitious that the pains which we are taking on behalf of the suffering poor should be better supported by the mass of our countrymen.

Last year, exclusive of the three great endowed hospitals of London, 2,000 beds remained empty throughout the year. Hospital Sunday last year brought in about £40,000, as against £27,000, the sum collected two years ago, so that there was an increase of £13,000. Supposing that this year we make up the same amount as last year, we shall make up only half towards the £80,000 wanted. I appeal to your sentiments of humanity, and to your sympathy with the suffering poor, to help our fellow-creatures. This is the year of Jubilee: I have had letters from at least some 500 towns and villages, asking for subscriptions, and you, too, doubtless have had to give subscriptions towards public festivals and entertainments, etc. It is those who are well who will enjoy themselves. Those who are ill will have to remain in their hovels, while the shouts of those who are enjoying themselves will reach their suffering ears. I am aware of the great claims which are made upon all classes in this Jubilee year. But all can spare something, and gifts shonld be gladly sent to the hospitals.

Now, in a year of general happiness and general jubilation like the present, I would ask the ladies if they cannot kindly put by a few of the pounds they would otherwise spend on making themselves more irresistible still, and bestow them on the hospitals, which are so sorely in need of subscriptions. They will, I am sure, be glad of having done so in the long run. I also appeal to the ladies to exercise their enormous influence with us, the "weaker" sex. I know the influence they can wield to make their husbands and their brothers subscribe towards our hospitals. And if I venture to appeal to them especially, it is for the great part which women themselves are taking in the success of the hospitals. I do not know if many of you have ever visited a hospital. I have the honour of being president of one, and I have never ceased wondering at and admiring the sympathy of the nurses, that noble army of women who have followed in the steps of Florence Nightingale. Many a time I have stood in one of the wards, and seen one of the nurses lull to sleep an agonised child, and I have shrunk away from the ghastly wound of an adult while the nurse has devotedly dressed it without showing the slightest disgust. I ask everybody, therefore, to use all the means in their power to aid the Hospital Sunday Fund to help our hospitals.

